# Response to ‘Jangle fallacy epidemic in obesity research: a comment on Ruddock *et al.* (2017)’

**DOI:** 10.1038/ijo.2017.290

**Published:** 2018-02-06

**Authors:** H K Ruddock, P Christiansen, J C G Halford, C A Hardman

**Affiliations:** 1 Department of Psychological Sciences, University of Liverpool, Liverpool, UK; 2 UK Centre for Tobacco and Alcohol Studies, Nottingham, UK

We thank Vainik and Meule (2017)^(ref. 1)^ for their comments regarding the validation of the Addiction-like Eating Behaviour Scale (AEBS). Drawing upon correlations observed between the AEBS and other measures of eating behaviour, Vainik and Meule suggest that the AEBS may contribute to ‘jangle fallacy’ (that is, the use of different questionnaires to capture the same construct) within obesity research. We are similarly mindful of this issue and agree that further discussion is important in order to advance research into addiction-like eating and obesity.

As Vainik and Meule point out, the two-factor structure of the AEBS (that is, appetitive drive/dietary control) reflects other measures of eating behaviour. Indeed, if we are to conceptualise addiction-like eating as an exaggeration of our natural motivation to obtain food, then it is not surprising that some items map onto existing questionnaires. Notably, recent research has shown that many eating behaviour questionnaires measure common underlying constructs of ‘uncontrolled eating’/‘food responsivity’ and ‘dietary restriction’,^2, 3^ and the two-factor structure of the AEBS is consistent with this. The AEBS may therefore be used as a single questionnaire that captures core eating behaviours that are associated with having higher body mass index (BMI).

The core behavioural processes captured by the AEBS are also extant in drug use, problematic drinking and other compulsive behaviours. Critically, the two-factor structure of the AEBS is entirely consistent with established dual-process theoretical models, which underpin a range of motivated behaviours (for example, eating, drug/alcohol use).^4^ Our analyses suggest that the AEBS specifically captures these ‘addiction-like’ processes. In our paper, AEBS and Binge Eating Scale (BES) scores differentially converged with measures of disordered eating and problematic drinking; AEBS scores correlated positively with problematic drinking but, unlike the BES, did not correlate with a measure of disordered eating (characterised by weight concern and dietary restriction). This suggests that the AEBS captures eating behaviours that share similar risk factors with other addictive disorders (that is, problematic drinking), and which are distinct from traditional eating disorders. This is important as the aetiology of compulsive overeating likely differs between individuals;^5^ while some individuals may engage in overeating following chronic attempts at dietary restriction, others may be driven by addiction-like processes towards food. Our findings suggest that the AEBS may usefully distinguish between subsets of individuals who engage in compulsive overeating.

To further establish the distinctiveness of the AEBS, it is important to examine the extent to which it predicts observable outcomes (for example, BMI) over existing measures of compulsive overeating (that is, incremental validity). To do this, Vainik and Meule suggest using a structural equation modelling framework using the scales’ latent variables. This approach provides a more reliable estimate of incremental validity (compared to the regression analysis reported) by controlling for measurement error.^6^ We are grateful for this suggestion and we have used this method to re-examine the scale’s ability to predict variance in BMI after controlling for the latent BES^7^ and Yale Food Addiction Scale (YFAS) ‘symptoms’.^8, 9^ Consistent with our reported findings, the AEBS significantly predicted variance in BMI when controlling for the BES and YFAS (and measurement error in the latent variables) (*B*=1.82, s.e.=0.76, *P*=0.017). These findings provide further support for the ability of the AEBS to capture behaviours that are not already accounted for by existing measures of compulsive overeating.
